# Resistance Feedback of a Ni-Ti Alloy Actuator at Room Temperature in Still Air

**DOI:** 10.3390/mi15040545

**Published:** 2024-04-18

**Authors:** Francesco Durante, Terenziano Raparelli, Pierluigi Beomonte Zobel

**Affiliations:** 1 Department of Industrial and Information Engineering and Economy (DIIIE), University of L’Aquila, P.le Pontieri 1, Località Monteluco, 67100 L’Aquila, Italy; pierluigi.zobel@univaq.it; 2 Department of Mechanical and Aerospace Engineering (DIMEAS), Politecnico di Torino, Corso Duca degli Abruzzi 24, 10129 Torino, Italy; terenziano.raparelli@polito.it

**Keywords:** shape memory alloys, control, electrical resistance feedback, soft actuator, Ni-Ti, room temperature

## Abstract

This paper illustrates an experimental activity for the closed-loop position control of an actuator made using shape memory alloy (SMA) wire. A solution with the self-sensing effect was implemented to miniaturize the systems, i.e., without external sensors. A proportional control algorithm was initially used, demonstrating the idea’s feasibility; the wire can behave simultaneously as an actuator and sensor. An experimental investigation was subsequently conducted for the optimization of the developed actuator. As for the material, a Flexinol wire, Ni-Ti alloy, with a diameter of 0.150 mm and a length of 200 mm, was used. Preliminarily, characterization of the SMA wire at constant and variable loads was carried out; the characteristics detected were elongation vs. electric current and elongation vs. electrical resistance. The control system is PC based with a data acquisition card (DAQ). A drive board was designed and built to read the wire’s electrical resistance and power it by pulse width modulation (PWM). A notable result is that the actuator works with good precision and in dynamic conditions, even when it is called to support a load up to 65% different from that for which the electrical resistance–length correlation has previously been experimentally obtained, on which the control is based. This opens up the possibility of using the actuator in a counteracting configuration with a spring, which makes hardware implementation and control management simple.

## 1. Introduction

Shape memory alloy (SMA) actuators have recently gained significant attention due to their unique capabilities in converting heat into mechanical energy. Their ability to recover their original shape after undergoing substantial deformation makes them suitable for use as actuators. Many works in the scientific literature propose using SMA as actuators [1,2,3,4,5,6,7]. Many scientific works concern their applications in many fields, such as micro-systems [8,9,10,11], robotics [12,13,14,15,16,17,18,19], biomedical [20,21,22,23,24,25], wearables [26,27,28,29], and aerospace [30,31,32,33,34].

However, miniaturizing SMA actuators poses several challenges, including the need for precise control, compact design, and improved performance. One of the key factors influencing the performance of SMA actuators, in the form of wires, is their possibility to perform position control since, traditionally, they are used as on–off actuators. Position control is not easy in the open loop since the SMA deformation behavior is affected by the actual percentage of phase transformation from martensite to austenite, which depends on mechanical stress and the actual temperature affected by energy, thermal capacity, and heat transmission [35,36,37]. On the other hand, the closed loop, although a robust method for the position control of actuators, needs additional hardware, such as sensors, to monitor the actual position and make it possible to send feedback to the controller. Nevertheless, sensors can be bulky and, in the case of our interest, prohibitive. For example, if we consider an SMA wire with a diameter of 0.15 mm, which is capable of a pull force of 3.3 N, which could potentially be a tiny actuator, it will lose its smallness if associated, for example, with a potentiometer. This was carried out in [38], where SMAs were implemented as actuators in a three DoF small parallel robot where each actuator was made using a 150 µm diameter SMA wire. A resistive potentiometer, which has dimensions many times larger than the actuator’s, was used for position control for each GDL; the ideal would be to have a sensor as small as the actuator.

Resistance feedback-based control techniques have emerged as a promising approach for enhancing the performance of miniaturized SMA actuators to address this challenge. By monitoring the resistance of the SMA wire or film during actuation, feedback control can be implemented to achieve higher precision and faster response times. This approach enables better control of the actuation force, position, and speed while improving efficiency. As mentioned above, for shape memory alloy wires, the deformation depends on the percentage of phase transformation, a function of the actual temperature that depends on many variables. However, it is possible to identify a link between the deformation and the electrical resistance as a bijection function (self-sensing effect). A link of this type can be used to acquire an estimate of the current length of the actuator. In these conditions, an SMA wire can perform the functions of the actuator and sensor at the same time.

Only some scientific works are related to implementing closed-loop SMA actuators using the self-sensing effect. Most implement the actuator drive through an analog system; these systems need, at the controller level, a DAC and, in the power section, an operational amplifier [39,40,41,42,43]. Although PWM control is frequently used to control general actuators, it has been seldom studied for SMA wire actuators with self-sensing effects. The authors of [44] proposed such a system by implementing proportional control. This approach is very convenient given that it requires straightforward hardware, which is present even on microcontrollers of minimal dimensions, as a digital port is sufficient. Meanwhile, the DAC, for processing analog signals, is a more sophisticated component and is only sometimes present on microcontrollers. Even a simple transistor can perform the power function, while an operational amplifier is needed with analog signals. To the authors’ knowledge, the same control approach, using the self-sensing effect via a PWM signal, is proposed in two other works. In these works, the model linking the actual deformation to the electrical resistance is expressed in a mathematical form depending on seven [45] or eight [46] coefficients.

The objective of the present work is to develop a procedure for implementing an actuator based on shape memory alloy wires with the self-sensing effect. The proposed process is in a simpler form than documented in the scientific literature. The procedure involves the use of a test bench specifically developed for the experimental characterization of the behavior of the wires. In particular, the bench allows for the determination of a mathematical model that correlates the deformation of the wire with the electrical resistance necessary for the operation of the actuator. In this work, the use of a simple (linear) model is documented, while in other works, a polynomial model with six or seven coefficients is used. This approach, together with the type of control, based on a PWM signal (for which an experimental investigation was carried out to determine the optimal operating parameters), makes the actuator implementable in applications using very simple and economical hardware components. In fact, for control, a microcontroller with a simple digital gate and, for the power part, a simple transistor are sufficient. This is unlike the systems documented in the literature which use an analog–digital converter for control and an analog current amplifier for the power section.

Once the procedure for the actuator was developed, proportional control was implemented. Subsequently, optimization was carried out through experimental tests aimed at highlighting the influence of different components of the control logic, i.e., proportional (P), derivative (D), integral (I), and their combinations. Also, the parameters of the PWM command and the influence of the acting antagonist force were investigated.

## 2. Materials and Methods

### 2.1. Shape Memory Alloy Wires: Experimental Characterization

In shape memory actuators, it is possible to implement feedback control without using dedicated sensors by exploiting a characteristic of these alloys known as the self-sensing effect, which allows you to link the deformation of the actuator to its electrical resistance.

**Figure 1:**
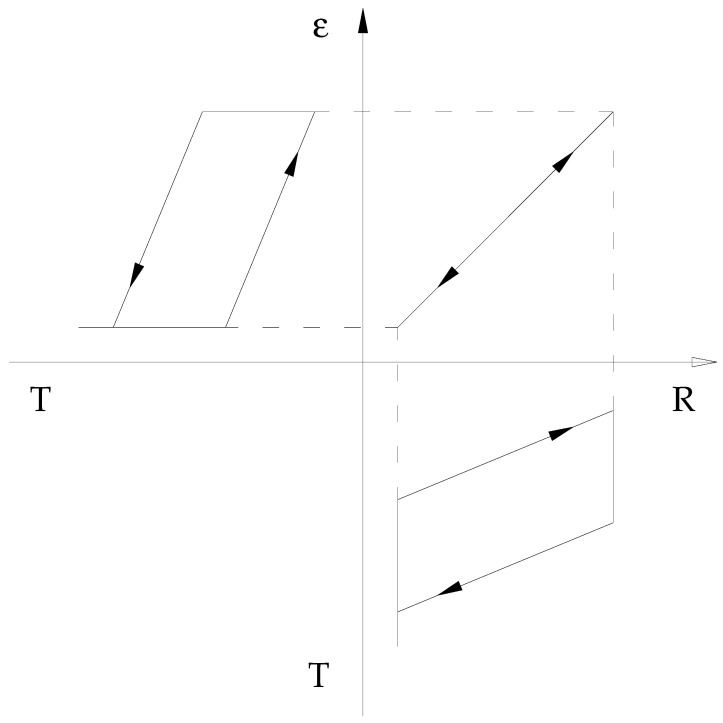
Deformation–resistance, resistance–temperature, and deformation–temperature characteristics for a Ni-Ti wire showing cubic to monoclinic transformation (not involving the R-phase).

The resistance vs. length trends, with respect to temperature, show strong hysteresis; on the contrary, the relationship that binds them together is almost free from this phenomenon, and if we exclude the two extreme phases of the transformation of the material (completely martensite or austenite), it can theoretically be considered linear (Figure 1).

To understand the phenomenon described, various experimental characterizations were carried out on shape memory alloy wires to identify a link between deformation and other measurable functional parameters to estimate the current deformation of the wire starting from the measurement of other variables. In particular, constant load and variable load strain tests were conducted in which strain and electric current were measured. Subsequently, another test campaign aimed to determine the link between deformation and the wire’s electrical resistance under constant and variable load.

#### 2.1.1. Test Bench

The thread under test is a commercial one, Flexinol brand, type 150 HT (Dynalloy, Irvine, CA, USA), whose characteristics are shown in Table 1, with a length of 200 mm.

**Figure 2:**
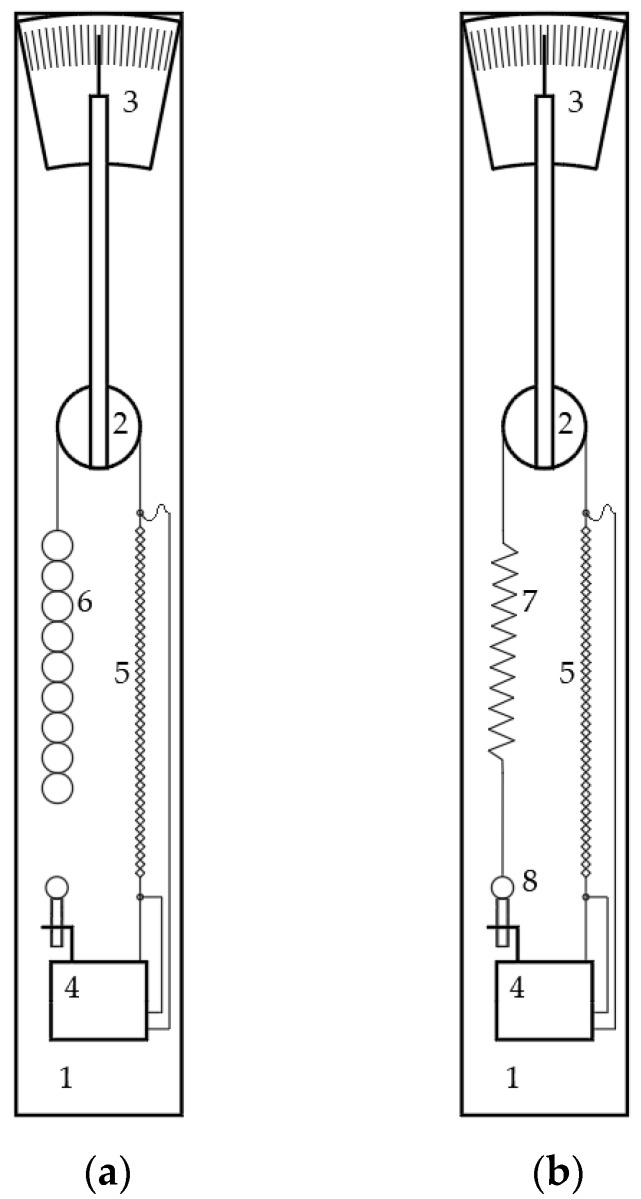
Test bench: arrangement for antagonist force by weights (**a**) and arrangement for the antagonist force by a spring (**b**). The test bench is composed of a frame (1), a pulley (2) as a return for the application of the load, which is connected to the index indicating the actual deformation on the graduated scale (3), the base (4) supporting the electric connections and the lower actuator fixing, the SMA wire actuator (5), and the weights (6) for the constant load tests (**a**). For the variable load configuration (**b**), as antagonist load, a spring (7) is used, the preload adjustment of which is possible to carry out by a screw (8).

To determine the deformation of the wire, a test bench was created in two versions, “a” and “b”, with “a” for the constant load tests and “b” for the variable load ones. With reference to Figure 2, the test bench consists of an upright frame (1) made by an aluminum profile with a rectangular section, measuring 80 mm × 40 mm × 4 mm and a height of 600 mm, on which different components are connected, that is:

- A pulley (2) that serves as a return for the application of the payload and to which an index for reading the movement of the free end of the wire is connected jointly;
- A graduated scale (3);
- A base (4), where the electrical connections and lower wire fixing are placed.

On the test bench, the SMA wire (5) is mounted between the base (4) and a Kevlar cord engaged in the pulley (2).

In the case of the constant weight load tests, the Kevlar cord is connected on the other end to some weights (6).

In the case of the variable load tests carried out with the opposition of a spring, the Kevlar cord is connected, on the other end, to a spring (7) suspended by the base (4) through a fixing system with a screw that allows for preload adjustment (8).

The mechanical connections between the components are made through eyelets and mini hooks; in particular, the eyelets are attached to the wire by the manufacturer’s “clamps”. Both the eyelets and the hooks are made of metal; in this way, it was possible to make the electrical connections on the hooks, allowing for easy wire replacement. The material of the connecting cord was chosen due to its mechanical properties in the length direction and its low resistance in the transverse direction; in particular, the specific tensile modulus of elasticity (modulus of elasticity/density) is more than three times that of steel. These features protect from any cord influences on the measuring chain. The pulley transforms the linear displacement of the free end of the wire into index rotations. At the same time, the graduated scale was divided to read the linear displacement of the wire directly. The entire measurement chain was designed in such a way as to have an amplification of displacement of a factor of 10. Since the system has been proven to be sensitive to external conditions, particularly air currents, everything was inserted inside a transparent Plexiglas tube. In the following paragraph, the experimental characterization of the SMA wire is described. All of the test sessions were conducted in almost stationary conditions, the power supply voltage was varied in small “steps”, and system stabilization was accounted for with each variation. The tests were conducted at the room temperature of 22 ± 2 °C. With these room temperature variations, there proved to be no relevant effects on the results.

#### 2.1.2. Electric Current–Strain Curves

**Figure 3:**
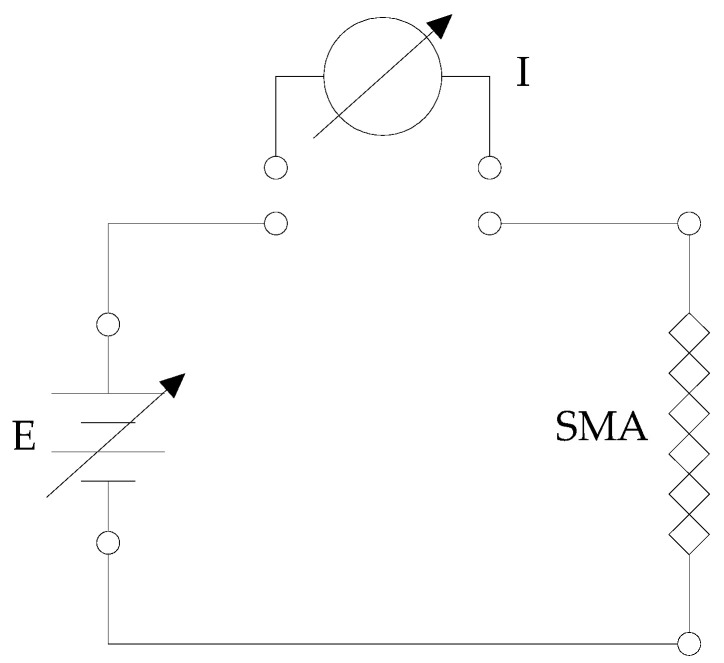
Measurement circuit.

The first investigations helped to determine the sensitivity, have a complete picture of shape memory alloys’ behavior, and evaluate the possibility of creating simple open-loop control systems. This activity led to the measurement of a series of current–strain curves obtained at constant and variable loads. The circuit in Figure 3 was created through which it was possible to vary the voltage applied to the ends of the wire and to read the intensity of the current flowing through it.

Using this circuit, together with the bench in Figure 2, allowed us to derive the link between the deformation of the wire and the current flowing through it. For ease of use, the experimental data are reported as a function of percentage shortening (ε%):

(1) $$\varepsilon \%=\frac{shortening}{initial\;length}\times 100$$

##### Constant Load Characterization

**Figure 4:**
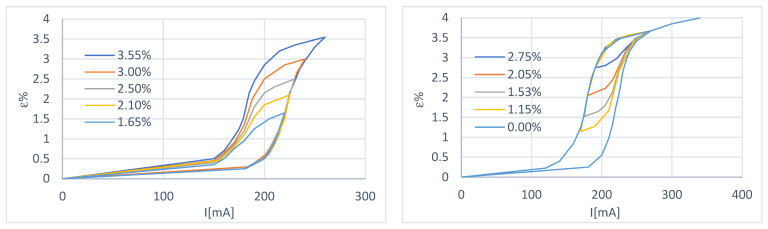
First-order sub-cycles of the deformation–current characteristic at constant load. Starting from an initial state of 100% martensite (**left**) and starting from an initial state of 100% austenite (**right**).

In this case, the payload consists of weights (the curves were obtained with a total load of 1.50 N), limiting the recovery of the actuator to a value equal to 3.5%. Two of the curves obtained are shown in Figure 4.

##### Variable Load Characterization

**Figure 5:**
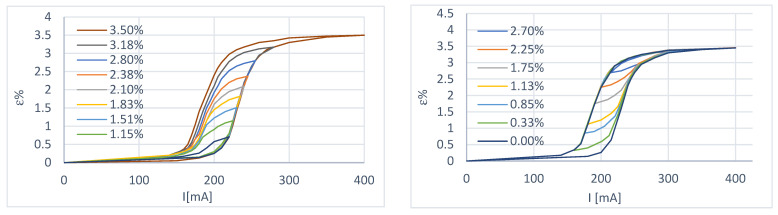
First-order sub-cycles of the deformation–current characteristic at variable load. Starting from an initial state of 100% martensite (**left**) and starting from an initial state of 100% austenite (**right**).

**Figure 6:**
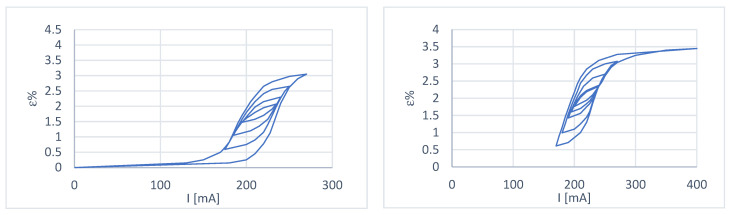
Multiple sub-cycles of the deformation–current characteristic at variable load. Starting from an initial state of 100% martensite (**left**) and starting from an initial state of 100% austenite (**right**).

Depending on the characteristics of the wire provided by the manufacturer, the tests were carried out in such a way as to have a maximum deformation of 3.5%, with a maximum load of 3.3 N and a recovery load (minimum) of 0.70 N. The above values were adjusted for the spring preload. The results are shown in Figure 5 and Figure 6.

**Figure 7:**
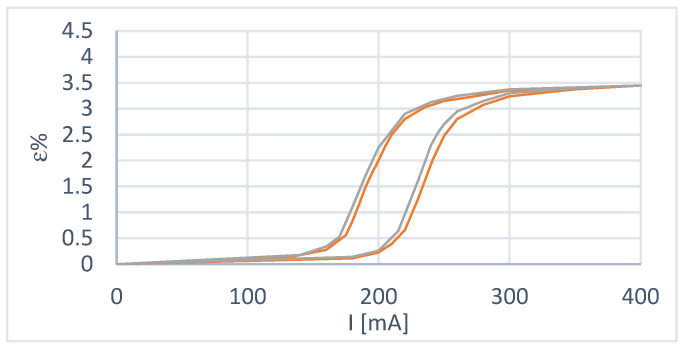
Comparative analysis between the two variable load test sessions.

Some considerations can be made from the curves obtained:

1. In the case of constant load at a deformation recovery value of 3.5%, the wire has yet to reach the condition of complete austenitization; this is evidently due to the more significant deformation to which it is subjected during cooling. In fact, with the use of the spring, it is possible to choose the extent of the deformation with an appropriate adjustment of the preload, while in the case of constant load, there is a return load (in our case, this is the paying load) that is more significant than the recovery load of the wire and this causes over-deformation;
2. The transformation interval is wider in the case of variable load because a deformation recovery value of 3.5% corresponds to a load of 3.3 N, which is more significant than in the case of constant load tests. This shows the dependence of the amplitude of the transformation interval on the applied load;
3. There is excellent repeatability of the behavior when moving on the primary hysteresis cycle;
4. Reaching a position starting from a condition other than 100% austenite or 100% martensite becomes complex (Figure 6);
5. Figure 7 shows two primary hysteresis cycles obtained at different times; we note the translation of the curve due to the different environmental conditions (different external temperatures).

One thing not visible in the curves is the strong sensitivity of the system to the presence of air flows.

#### 2.1.3. Electric Resistance–Strain Curves

**Figure 8:**
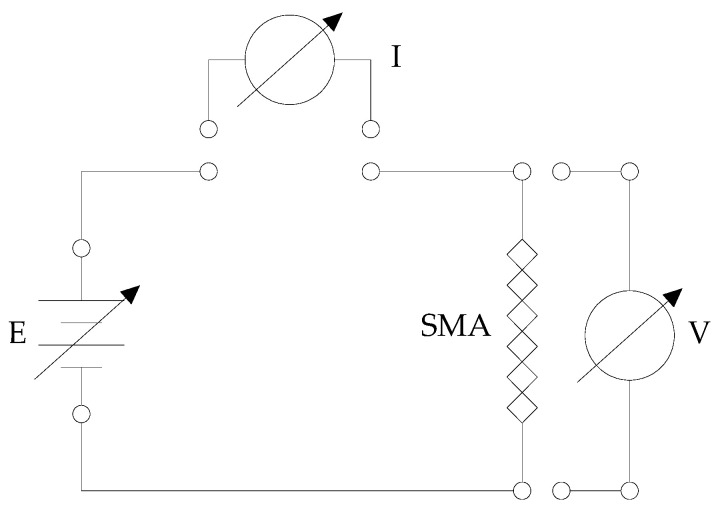
Circuit for determining the electrical resistance of the SMA wire.

These curves were obtained using the test bench in Figure 2 and the circuit in Figure 8. Resistance was calculated with Ohm’s law (R = V/I) by directly reading the values of the current passing through the wire and the potential difference established across it.

##### Variable Load Characterization

**Figure 9:**
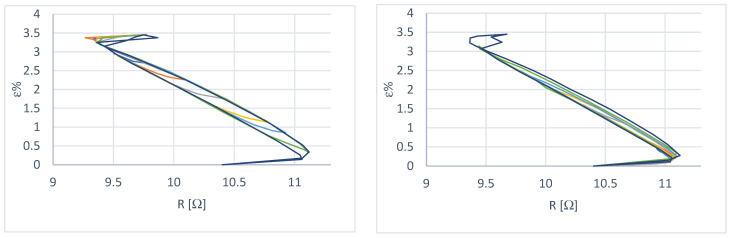
First-order hysteresis sub-cycles with variable load starting from a state of 100% austenite (**left**) and starting from a state of 100% martensite (**right**).

The deformation–resistance characteristic (ε-R) was obtained under variable load conditions, following the procedure illustrated to determine the ε-I curves. Two results from the test sessions are shown in Figure 9.

##### Constant Load Characterization

**Figure 10:**
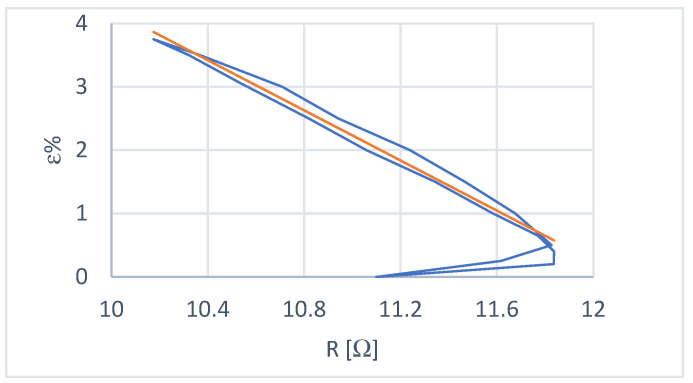
Constant load characteristic and trend line.

The constant load deformation–resistance characteristic, shown in Figure 10, was obtained with a load of 2.0 N. It also shows the trend line obtained by excluding the extremes of transformation. The load of 2.0 N represents a possible payload supported by the actuator.

The data obtained show that:

1. The ε-R characteristic is not free from hysteresis phenomena. The amplitude of the cycle is significantly lower than that of the ε-I characteristic, however;
2. The mean curve of the cycle, excluding the extremes of the transformation, can be considered linear;
3. The data are characterized by good repeatability on the external hysteresis cycle;
4. Providing a one-to-one deformation–resistance relationship at the extremes of the transformation interval is impossible.

### 2.2. Control with Self-Sensing

From the above data, it can be concluded that the current-deformation curves cannot be used to estimate the actual length of the wire since the marked hysteresis and the abrupt trend of the graphs, which is non-linear, make it impossible to identify a precise value of the deformation starting from the electric current flowing through the wire. At the same time, the characteristic electrical resistance–deformation presents a more gradual, linear trend and a significantly reduced hysteresis. From the graphs, both at variable and constant loads, it is immediately evident that it is possible to identify a one-to-one correspondence between electrical resistance and deformation for a large section of the graph. These curves were used to implement actuator position control. In particular, position control with constant load was implemented. The idea was to create a control cycle that provides, in succession, the power supply to the actuator to make it contract and, subsequently, to interrupt the power supply for a very short time to measure the actual electrical resistance to rise through the detected characteristic to the actual length and implement feedback in a continuous cycle. The SMA wire was thus found to alternatively perform the functions of the actuator and the sensor.

**Figure 11:**
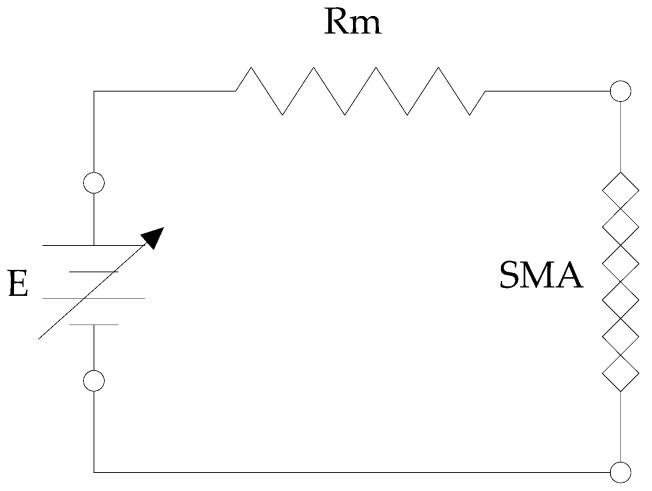
Wire electrical resistance measurement circuit diagram.

Power is delivered via a pulse width modulation (PWM) signal; the electrical resistance of the actuator was determined by reading the voltage drop across the SMA wire and a known resistance according to the scheme in Figure 11.

The resistance of the SMA wire was estimated with the relationship:

(2) $$Rsma=\frac{Vsma}{Vm}\cdot Rm$$

The model for estimating the actual deformation from the electrical resistance is in (3).

ε = 0.043 − 0.00804 R(3)

#### 2.2.1. Control Architecture

**Figure 12:**
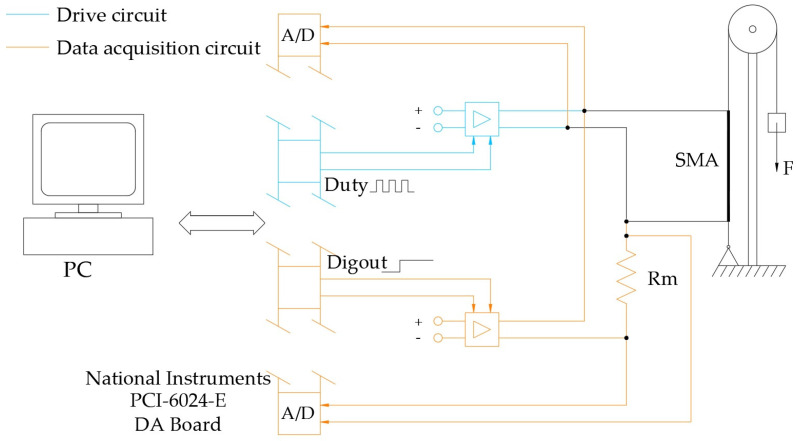
Functional diagram of the control system.

Figure 12 shows a block diagram of the control system.

There are four parts:

1. The characteristics of the actuator (the SMA wire) are shown in Table 1;
2. The drive is the power circuit equipped with the electrical and electronic components that allow the actuator to be powered;
3. The measurement circuit is based on the diagram in Figure 11, which determines the length of the actuator;
4. A PC, in which the control algorithms reside, with a National Instruments PCI-6024E (National Instruments, Austin, TX, USA) data acquisition card for interfacing the PC and drive and measurement unit.

**Figure 13:**
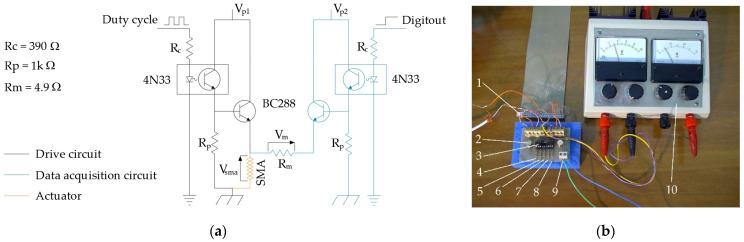
Board used for the measurement of electrical resistance and driving of the actuator: (**a**) the electric scheme and (**b**) the physical homemade board: (1) controller connector, (2) BC288 transistor, (3) 4N33 optocoupler, (4–8) R_c_ R_p_ R_m_ electrical resistances, (9) Ni-Ti actuator connector, (10) and the power supply.

In the embodiment, the drive and measurement circuits are mounted on a homemade board but constitute two independent sections (Figure 13).

Each of the two circuits consists of

- A 4N33 optocoupler used to separate, from an electrical point of view, the control part from the power part to avoid possible damage to the data acquisition card;
- A BC288 transistor which enables transition of the power signal;
- A polarization resistance (Rp);
- A resistance on the control branch (Rc) used to lower the voltage across the optocoupler;
- On the measurement circuit, there is the resistance Rm, which is necessary to determine the resistance of the wire in the SMA.

The value of the polarization resistance (Rp = 1 kΩ) was chosen according to the optimization of the circuit response times, and the value of 1 kΩ was adopted, as it also allows one to limit the current in the optocoupler.

The PWM signal for enabling the passage of power to the actuator has a base frequency of 30 kHz, and the power circuit has a response time of less than one ten-thousandth of a second.

#### 2.2.2. Control Algorithms

**Figure 14:**
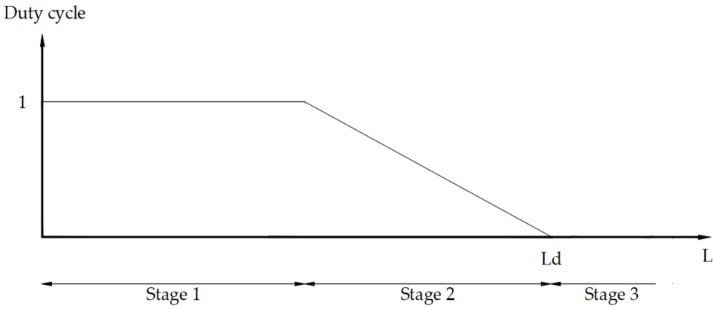
Control logic scheme: in stage 1, far from the target, the control supplies the maximum power. In stage 2, close to the target, the control becomes proportional. In stage 3, overcoming the target, the control cuts power off.

The explanatory scheme of the control logic adopted is visible in Figure 14.

Three phases are recognized:

1. When the actuator is far from the desired deformation value (phase 1), the control sends the maximum possible power (duty cycle = 1);
2. When the actuator is close to the desired deformation (phase 2), the control becomes proportional (P);
3. If the actuator exceeds the desired shortening (phase 3), the power supply is cut off, and the recall load provides for the deformation (elongation) of the actuator.

This control logic allows a faster approach phase than a purely proportional one.

To read the voltages with which to trace the deformation of the actuator, the power and measurement circuits are powered alternately according to the following cyclic sequence:

1. The power circuit is activated;
2. The power circuit is deactivated;
3. The measurement circuit is activated;
4. The voltages are read;
5. The measuring circuit is deactivated.
6. Phases 3–5 have an overall duration equal to 2.2% of the total operating time.

#### 2.2.3. Experimental Validation

To conduct the experimental tests, a measurement chain was created, which supports the developed system and allows one to measure the displacements of the actuator and compare them with the desired ones. It consists of:

- A National Instruments PCI-6034E data acquisition card;
- A PC where the card management program resides;
- A position transducer.

**Figure 15:**
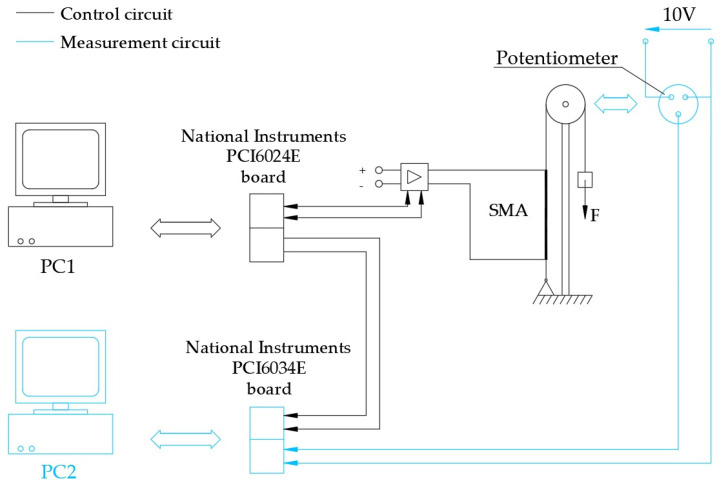
Experimental setup scheme.

Figure 15 shows the experimental setup scheme.

The measurement sensor is a servo potentiometer suitable for use with precision DC motors or as a position transducer. The contacts are multi-brush and guarantee a high resolution. Two bearings reduce friction to a minimum and require a low torque for shaft rotation. Table 2 shows the characteristics of the potentiometer.

**Figure 16:**
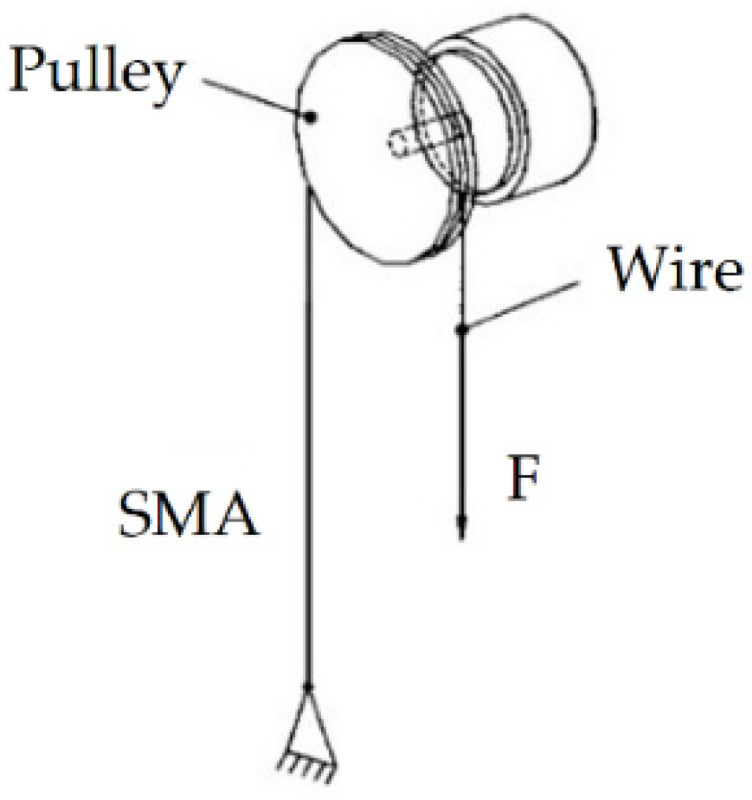
The pulley mounted on the axis of the potentiometer.

The pulley for the recovery load was mounted on the axis of the potentiometer (Figure 16).

The power supply was set to 10 V to make the most of the transducer’s available sensitivity. The Vinit values (voltage supplied by the potentiometer in zero conditions) and the proportionality constant of the transducer (Kt) are shown below:

- Vinit = 3.127 V
- Kt = 0.1 V/mm.

The control program of the board provides for the timed reading of the voltage value of the potentiometer at regular intervals of 0.1 s. The card is enabled for reading through a consent signal sent by the wire control system, and the connection is made through a digital channel. When the board detects voltage on this channel, it starts reading the potentiometer, which ends when the signal returns to zero. The consent signal allows the reference and position signals to have the same time base.

## 3. Results

### 3.1. Performance with Proportional Control

**Figure 17:**
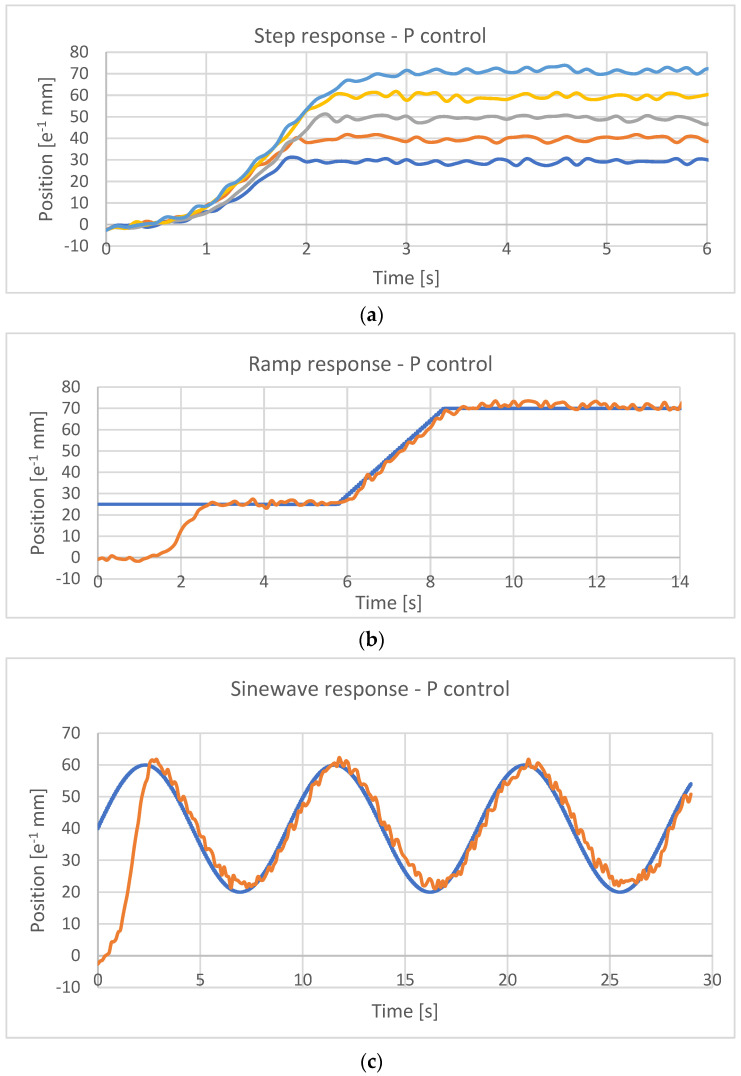
Response to several functions: (**a**) step responses for the target ranging from 0.3 mm to 0.7 mm; (**b**) ramp responses for a target of 0.7 mm; and (**c**) responses for a sinewave with the mean value of 0.4 mm, an amplitude of 0.2 mm, and a period of 9 s.

Experimental tests were conducted for the real-time control of the position of the developed actuator. Performance was investigated for step- (Figure 17a), ramp- (Figure 17b), and sinusoidal-type inputs (Figure 17c).

The onset of vibrations around the reference position reached by the actuator can be noted despite good behavior in reaching the assigned position.

The same behavior type was found in variable references such as the ramp or the sinusoidal trajectory.

### 3.2. Performance after Optimization

The need to obtain more stable positioning around the desired position led to analyzing the causes of the vibrations and identifying possible solutions according to those parameters that could significantly influence the system.

All those variables, from the control logic to the various functional parameters that could improve the response, were considered.

Attention was mainly focused on three aspects that primarily seemed to affect the behavior of the system:

- The control logic;
- The actuator power signal (PWM);
- The return force exerted on the wire.

Tests were conducted by varying each of the parameters described above from time to time to identify the possible optimal combinations that would improve the performance of the control.

As regards the control logic, it was decided to move from purely proportional to proportional–integral–derivative (PID) control and also to experiment with the behavior of the different combinations: proportional–derivative (PD) and proportional–integrative (PI).

We also worked on the signal that feeds the PWM SMA wire, having noticed evident effects of the square wave frequency that characterizes the input signal. Also, in this case, proceeding experimentally, it was possible to identify the optimal values to be assigned to this parameter.

Finally, we intervened on the return force acting on the wire by applying weights of different values since, from preliminary tests, it was found that the variation in the force resistant to the movement of the actuator affects the quality of the system’s response.

As regards the calibration of the control parameters, we proceeded with the Ziegler–Nichols criterion, obtaining the values shown in Table 3, while in Table 4, the optimization experimental plan is presented.

The control system was tested for different types of input references:

- Step entrances;
- Ramp trajectory tracking;
- Sinusoidal trajectory tracking;
- Response to external disturbance.

**Figure 18:**
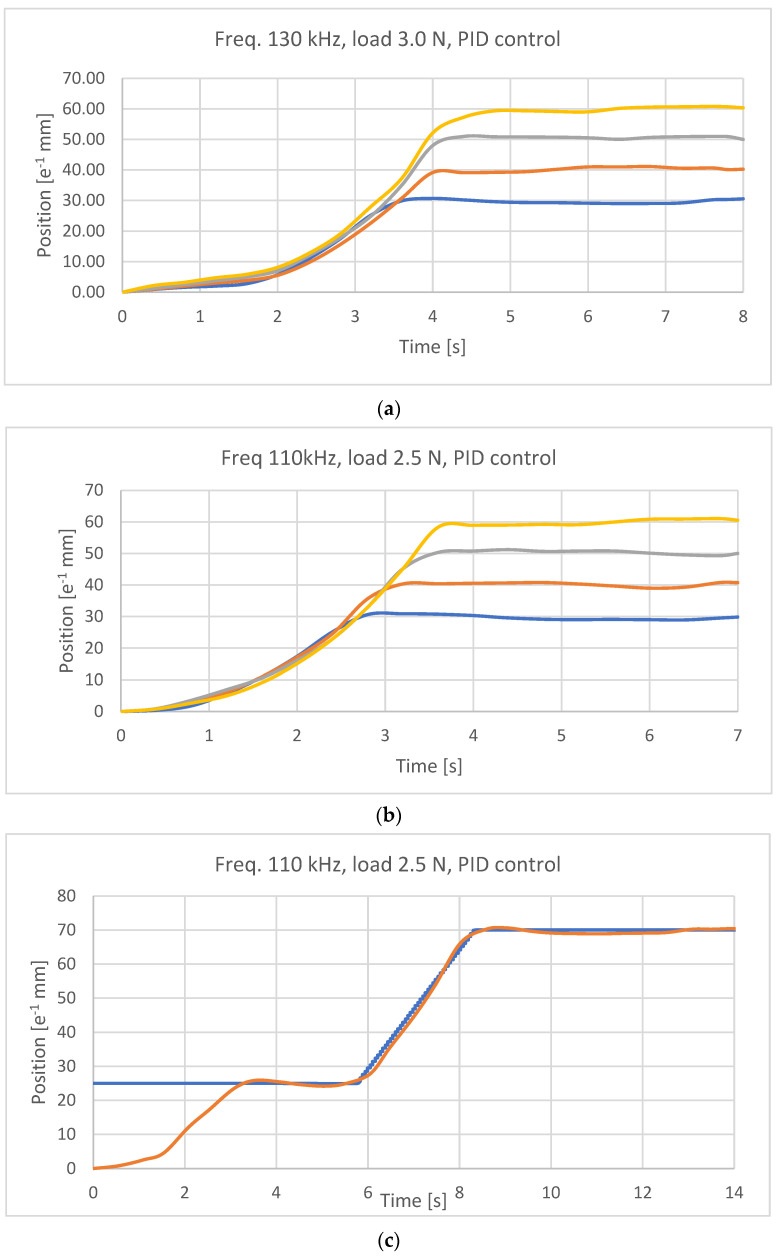

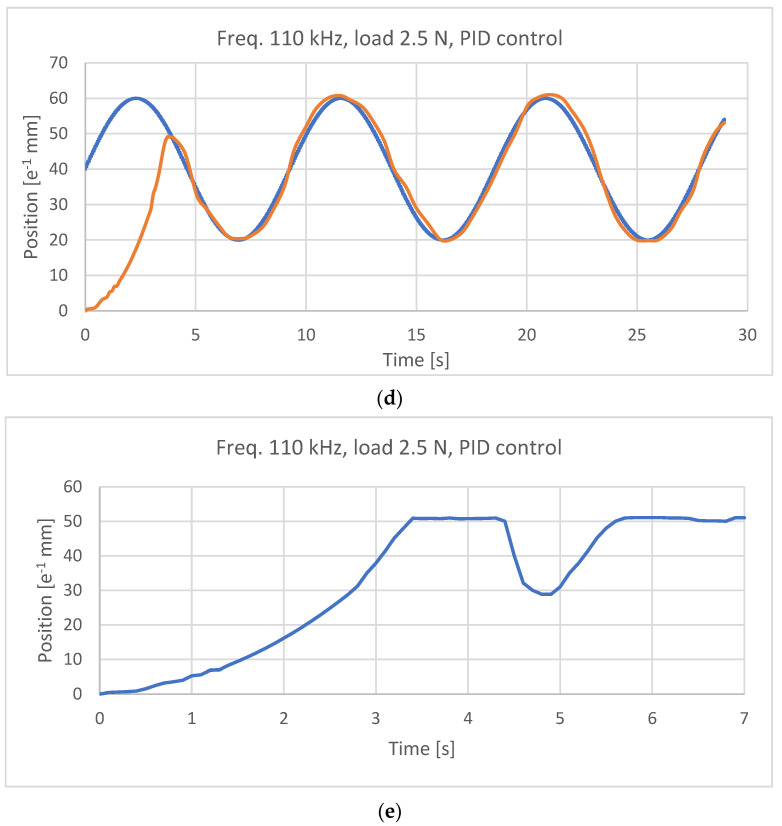
Response to several functions: (**a**) step responses for the target range from 0.3 mm to 0.6 mm for a combination of experimented parameters; (**b**) step responses for the target ranged from 0.3 mm to 0.6 mm after optimization; (**c**) ramp responses for a target of 0.7 mm after optimization; (**d**) responses for a sinewave with the mean value of 0.4 mm, an amplitude of 0.2 mm, and a period of 9 s after optimization; (**e**) response to a disturbance at 4.4 s with a 0.5 s duration and consisting of an air flow, at room temperature of 22 °C, causing, in the central part, the actuator commanded to achieve a deformation of 5 mm, with disturbance provided by a pneumatic tube with a diameter of 8 mm at a distance of 50 mm from the actuator, supplied by a pressure of 0.04 MPa.

Figure 18a presents the responses to step input for a combination of the tested parameters. In Figure 18b–d, the responses to step input, ramp input, and sinusoidal input for the optimized combination of the tested parameters are presented, respectively.

As for the effects of environmental temperature changes on control, only a few preliminary experiments were carried out. The effects of air flow, at room temperature (22 °C), applied on the actuator, through a pneumatic tube, were investigated. The actuator was commanded to produce a deformation of 5 mm (ε% = 2.5); the tube, with a diameter of 8 mm, was placed at a distance of 50 mm from the actuator and delivered an air flow through a ΔP equal to 0.04 MPa. The jet lasted 0.5 s.

In Figure 18e, the response to the disturbance described is shown. After the start of disturbance (at 4.4 s), a deviation of about 2 mm can be seen, from the reference already achieved, during the flow acting time interval. After about 0.8 s from the end of disturbance, the system achieves the reference again.

## 4. Discussion

To evaluate and compare the performance of the different systems as the configurations vary, the following indicators were identified:

- Max and min values reached in positioning;
- Fluctuation % = $\frac{Max-Min}{stroke}\cdot 100$;
- Average value.

By “stroke”, this means the maximum stroke of the actuator equal to 7.5 mm.

The values obtained are summarized in Table 5, Table 6 and Table 7.

Based on the experimental results, the following observations can be made. As for the PWM control signal frequency, this value was initially set to 30 kHz.

An increase in the frequency value corresponds to a less reactive response of the actuator once the desired position has been reached. By further increasing the value of this parameter, a situation is reached in which the system becomes insensitive to the control; the position reached is the full-scale position regardless of the set reference value.

The recovery force influences this parameter; with the initial value of 2.0 N, the contraction of the wire is very rapid. By increasing it, the frequency can be set to increasingly higher values. The limit value for the frequency of the control signal, applying a restoring force of 2.0 N, is approximately 100 kHz; with a force of 3.0 N, it is possible to reach frequencies of about 130 kHz. The maximum working recovery force that can be used relates to the yield strength of the Nitinol 150 HT wire, equal to 190 MPa, i.e., 3.3 N.

The increase in the counteracting force led to a reduction in the amplitude of the oscillations; at the same time, the system’s response times increased. With a weight of 3.0 N, there were delays in positioning of approximately 2 s. A good compromise between response times and vibration attenuation was achieved by applying weights of 2.5 N. It should be noted that although the system was not characterized according to the different weights applied, the same resistance-deformation curve was used, and the results obtained are still satisfactory.

Regarding the control type, a proportional–derivative control system was considered after implementing proportional control. However, control of this type cannot guarantee precise positioning, so an integrative component was added to eliminate the error when fully operational. Introducing a P.I.D control does not eliminate vibrations but is still advantageous for the good precision and robustness of the system. In particular, obtaining a good response for position values smaller than those achievable with the proportional system was possible. It was possible to reach the 20 position on the graduated scale, corresponding to a 2 mm shortening of the SMA wire.

The optimal combination of the parameters illustrated above was the following:

Square wave frequency: 110 kHz;

Recovery/applied force: 2.5 N;

Control type: PID.

Furthermore, the parameter that most seems to affect the unstable behavior of the SMA actuator is the frequency of the PWM activation signal.

## 5. Conclusions

This paper presents the development of an SMA wire actuator with feedback position control using the self-sensing effect for estimating the actual length of the actuator. From a hardware point of view, the system was implemented using electronic components with very simple operation thanks to the PWM-type control signal. A digital port at the controller level and a transistor in the power section are sufficient. As for the model linking the deformation of the wire to its electrical resistance, a model obtained experimentally was used. It can be expressed through a simple linear law.

The actuator can work in an agonist–antagonist configuration with weights or with a spring. Proportional-type control was implemented, and it presented oscillations. Subsequently, experimental research was carried out to find the optimal configuration considering the control logic components, the PWM command parameters, and the influence of the counteracting force. After parameter optimization, there was a reduction in the amplitude of the oscillations of approximately 35%. However, steady-state fluctuations were present, but positioning was carried out with an accuracy of below approximately 2.5%; the frequency was reduced from around 10 Hz to 1 Hz, resulting in much smoother behavior. The optimal configuration is the one in which PID control is implemented, with a PWM signal frequency equal to 110,000 Hz and a counteracting force equal to 2.5 N.

The result is an actuator well suited to miniaturized systems and even drivable by a tiny controller and driver.

## Figures and Tables

**Table 1:** Technical specification of the wire.

Flexinol 150HT
Wire diameter: 150 µm
Linear resistance: 50 Ω/m
Maximum recovery force: 10.4 N
Recommended deformation ratio: 3–5%
Austenite start temperature: 68 °C
Austenite finish temperature: 78 °C
Martensite start temperature: 52 °C
Martensite finish temperature: 42 °C

**Table 2:** Characteristics of the potentiometer.

Vishay 157
Maximum current on contacts: 10 mA
Electric rotation angle: 340 ± 4°
Mechanical rotation angle: 360°
Starting torque (max): 28 × 10^–4^ Nm
Torque at steady state (max): 21 × 10^–4^ Nm
Supply voltage (max): 10 V
Maximum electrical resistance: 10 kOhm

**Table 3:** Control parameters used in the experimental tests.

	K_p_	T_i_	T_d_	K_i_	K_d_
P	0.3				
PI	0.27	2.55		0.10	
PID	0.37	1.7	0.425	0.21	0.16

**Table 4:** Optimization experimental plan.

Control	Load	Rate
P	2.0 N	30 kHz
PI	2.5 N	110 kHz
PID	3.0 N	130 kHz

**Table 5:** Test with load: 2.0 N, rate: 30 kHz, and P control.

	Ref. 20	Ref. 30	Ref. 40	Ref. 50	Ref. 60	Ref. 70
Max [10^−1^ mm]	22.3	30.8	41.8	50.8	61.3	73.8
Min [10^−1^ mm]	17.6	27.4	37.9	46.5	56.9	69.6
Fluctuation %	6.2	4.5	5.2	4.5	5.8	5.6
Mean [10^−1^ mm]	19.7	29.1	39.8	49.4	59.4	71.4

**Table 6:** Test with load: 2.5 N, rate: 110 kHz, and PID control.

	Ref. 30	Ref. 40	Ref. 50	Ref. 60
Max [10^−1^ mm]	30.98	41.40	51.20	61.18
Min [10^−1^ mm]	29.32	38.96	48.96	58.87
Fluctuation %	2.21	3.25	2.99	3.08
Mean [10^−1^ mm]	30.38	39.81	50.16	

**Table 7:** Test with load: 3.0 N, rate: 130 kHz, and PID control.

	Ref. 30	Ref. 40	Ref. 50	Ref. 60
Max [10^−1^ mm]	30.87	40.80	51.02	61.13
Min [10^−1^ mm]	28.88	38.76	48.78	59.09
Fluctuation %	2.65	2.72	2.99	2.76
Mean [10^−1^ mm]	29.98	39.19	49.94	59.87

## Data Availability

The original contributions presented in the study are included in the article, further inquiries can be directed to the corresponding author.
